# The specialty choice of medical students in China: a stated preference experiment

**DOI:** 10.1186/s12909-016-0619-z

**Published:** 2016-04-12

**Authors:** Dong Liang, Cheng-Xiang Tang

**Affiliations:** Department of Health Management, School of Public Health, Fujian Medical University, Xueyuan Road, University Town, Fuzhou, Fujian 350108 China; Centre of Health Policy Research, Fujian Medical University, Xueyuan Road, University Town, Fuzhou, Fujian 350108 China; School of Public Administration, Southwestern University of Finance and Economics, Chengdu, Sichuan 610072 China

**Keywords:** Specialty choice, Best-worst scaling, China

## Abstract

**Background:**

Primary Care Providers (PCPs), such as internists and general practitioners, have been deemed a way of delivering cost-effective care in an equitable way because PCPs are responsible for providing accessible basic medical care for the general population. This study aims to examine medical students’ preferences for PCP-based specialty choices in the context of an ageing population in China.

**Methods:**

We implemented a Best-Worst Scaling (BWS) experiment, a recently developed preference elicitation method based on random utility theory. Three hundred and fifty graduating medical students from three medical colleges were randomly recruited to evaluate 11 common medical specialties in China. A counting approach, a conditional logit model, and K-means clustering have been used to analyse the relative importance of items and preference heterogeneity among medical students.

**Results:**

One hundred and ninety of 350 students completed valid questionnaires. General surgery was identified as the most preferred specialty among the overall sample, yet internal medicine shares the same importance as surgery. Both geriatric medicine and psychiatric medicine were found to be the least selected specialties. Finally, the K-means clustering further suggested there was preference heterogeneity across our sample.

**Conclusions:**

Two aims were fulfilled in this study. First, through our experimental approach the results provide a better understanding of the career desires of medical students in China. Second, the results of this study indicate that despite the fact a non-PCP-based specialty is the most popular among the sampled students; a PCP-based specialty is still an important alternative choice.

## Background

So far, very few studies have investigated the provision of health care professionals in China, even though the Chinese health care system faces big challenges, such as an ageing population and rapidly changing socio-economic influences [1]. On one hand, China has the largest ageing population among countries due to steep declines in fertility (e.g. the one-child policy) and an increase of life expectancy over the past 30 years [2]. On the other hand, rapid income growth has adversely affected daily activities causing a rising obesity and road traffic fatalities [3]. These changes call for a significant response from China’s health care professionals. This paper attempts to offer some important insights into the world’s largest education system for a health care workforce.

Complexities of the medical education system require us to understand perceptions and preferences of medical students, especially their willingness to choose PCP-relevant specialties in the future. However, we have found no present study in China that has ever tried to investigate these complexities using rigorous methods.

The reasons are extensive. First, China has a unique education system for training medical professionals. China’s system has been experiencing enormous transitions [4]. Nationwide the number of students enrolled in professional medical training increased four-fold from 2000 to 2012. Yet 17 % of medical students were assigned a medical specialty after their college entrance examination, instead of being offered a choice specialty [1].

Second, the health care system in China has been emphasising the role of hospitals, so PCPs such as general practitioners and internists are highly under-developed [5]. However as we know, using PCPs is an essential way of delivering equitable, cost-effective care to the general population [6].

Third, a crisis of violence against the medical workforce has also influenced many medical students’ choices on how to move their medical career forward [7]. Therefore, our study makes a major contribution in advancing our knowledge of China’s graduating medical students’ preferences for medical specialties, especially PCP-based specialties.

Our study employs a Best-Worst Scaling (BWS) experiment, a recently developed preference elicitation method based on random utility theory, to explore how students make decisions about choosing their medical specialty. The existing studies on relevant topics only adopt qualitative methods, such as focus group discussions, semi-structured interviews, Likert-style rating scales, or ranking tasks [8–11]. The BWS approach has clear advantages over other methods; for instance, BWS is a stated preference method, which has a sound theoretical basis in both economic and psychological fields [12]. Moreover, BWS has a relatively low cognitive burden. However, as a quantitative method of preference elicitation, BWS data provides rich information, which can be analysed by some advanced statistical models, for example, the conditional logit model, or the latent class model. To our knowledge, this is the first study using BWS to explore the medical specialty choices made by graduating medical students.

We conducted a BWS experiment to collect choice information about medical specialties from graduating medical students across three colleges in China. This study aims to address two gaps in the current literature: (i) To explore perceptions and preferences regarding the choice of a medical specialty through the application of a rigorous methodology; (ii) To investigate how graduating medical students in China value the PCP-based medical specialties.

## Methods

Researchers or policy makers are often interested in measuring a subject’s preference strength for a number of objects. BWS is one of the recently developed preference elicitation methods. It was initially introduced to assess public preferences for various aspects of food safety in 1992 [13]. BWS was further categorised into three types according to the nature of objects: the object item (case 1), the attribute item or profile case (case 2), and the multi-profile case (case 3) [14]. Since 2005, Best-Worst Scaling has gradually become more popular in health economics and health policy decision making because the formal mathematical proof of the Best-Worst probabilistic model underpinning the method was published at that time [15, 16]. In this study, we have conducted a case 1 BWS to measure how graduating medical students in China assess the value of medical specialties. The BWS experiment develops through the following stages: selection of medical specialties, experimental design and construction of choice sets, followed by survey implementation.

### Medical specialties selection

Based on a review of the literature and a focus group discussion with nine medical students, we developed a list of specialties, which can be chosen by medical students. At first, we drafted an initial list including 21 common medical specialties. Qualitative methods are recommended to develop the items used in a stated preference experiment. Thus, we conducted a focus group discussion to further refine the selection of specialties and develop a general wording for the questionnaire.

After a focus group discussion, we merged and selected 11 of the most cited medical specialties for three reasons:

(i) The sample was not targeted towards medical residents, therefore it was highly recommended to merge internal medicine, surgery and their associated sub-specialties, so they would be familiar and distinct for most of the medical students in college [17].
(ii) Defining PCP-relevant specialties is difficult in China, unlike some developed countries, for instance Switzerland, where specialists in either general medicine or internal medicine generally deliver primary health care [11]. Even though there is a serious shortage of trained general practitioners in the country, we did not include general practice in the list because it has been planned to develop as an academic discipline in universities since 2011 [18]. Following the previously established definition, we have classified internal medicine, paediatrics and geriatric medicine as PCP-relevant specialties [6, 19].
(iii) We have also excluded traditional Chinese medicine specialties because traditional Chinese medicine is still required to blend traditional medicine and modern science, and more importantly, it lacks popularity among medical students [20].

A brief description of all of the specialties is presented in Table 1:

**Table 1:** The 11 specialties for medical students’ choices

Specialties	Definition
Internal medicine	Provides long-term, comprehensive care in the office and in the hospital, managing both common and complex illnesses of adolescents, adults, and the elderly.
General surgery	Has principal expertise in the diagnosis and care of patients with diseases and disorders affecting the abdomen, digestive tract, endocrine system, breast, skin, and blood vessels. A General Surgeon is also trained in the treatment of patients who are injured or critically ill, and in the care of pediatric and cancer patients.
Ob & Gyn	Focuses on the health of women before, during, and after childbearing years, diagnosing and treating conditions of the reproductive system and associated disorders.
Paediatrics	A specialty is concerned with the physical, emotional, and social health of children from birth to young adulthood.
Ophthalmology	A specialty focused on the medical and surgical care of the eyes.
Otolaryngology	Provides medical/surgical therapy for the prevention of diseases, allergies, neoplasms, deformities, disorders, and/or injuries of the ears, nose, sinuses, throat, respiratory, and upper alimentary systems, face, jaws, and the other head and neck systems.
Oncology	Has expertise in the diagnosis, multidisciplinary treatment, and rehabilitation of patients with rare, uncommon, or complex cancers.
Geriatric medicine	A physician with special knowledge of the aging process and special skills in the diagnostic, therapeutic, preventive, and rehabilitative aspects of illness in the elderly
Dermatology	A specialty focused on the diagnosis and medical/surgical management of diseases of the skin, hair and nails, and mucous membranes.
Psychiatry	A psychiatrist specializes in the evaluation and treatment of mental, addictive, and emotional disorders such as schizophrenia and other psychotic disorders, mood disorders, anxiety disorders, substance-related disorders, sexual and gender identity disorders, and adjustment disorders.
Rehabilitation	A physician who evaluates and treats patients with physical and/or cognitive impairments and disabilities that result from musculoskeletal conditions (such as neck or back pain, or sports or work injuries), neurological conditions (such as stroke, brain injury, or spinal cord injury), or medical conditions.

### Experiment design and survey

Because of the nature of a constant comparison item size in each choice set, the Balanced Incomplete Block Design (BIBD) was preferred in the BWS. We chose a BIBD with a subset of 11 medical specialties assigned to each choice set based on a matrix. We have included the complete design matrix in Appendix A. The questionnaire introduced the basic definition of the specialties to the participants at first, regardless of their own understanding of the professions. Next, they were asked to choose their preferred specialty by imagining they had the chance to decide their own specialty in the future. Students were asked to indicate the most/least attractive specialty in each choice set. Each respondent was required to provide an answer to 11 choice sets in the final questionnaire. Each choice set consisted of five alternatives out of 11 specialties.

Between February and March 2015, 350 medical students from three medical colleges in the Fujian province in China, were randomly selected to answer the questionnaire. Approval to conduct the survey was obtained from each college. The data was collected based on a completely voluntary and anonymous principle. The Ethics Review Committee (ERC) of Fujian Medical University has granted us an exemption letter for ethical review considered the features of the study.

### Results analysis

The BWS scores, as the most common estimators for the BWS method, are calculated for each specialty. It has been proven that BWS scores are sufficient statistics for the likelihood function if we assume the maximum difference scaling (maxdiff) model in the statistical estimation [21]. In addition, we ranked the standard BWS scores for all of the specialties and presented the distribution of the BWS scores by specialty. Despite the above counting approach, we also employed a modelling approach using a conditional logit (CL) model to analyse the responses. To clarify the relative importance between the specialties, the coefficient or utility based on the CL estimation was converted into the share of preference for each specialty. Finally, we divided the respondents into two groups to investigate the preference heterogeneity across our sample by using K-Means clustering. This is a traditional cluster approach that involves minimising within-cluster variance and maximising across cluster variance.

## Results

Of the 350 graduating medical students who participated in the survey, 190 (54 %) completed and returned valid questionnaires. Among the valid sample, over half of the respondents were female (55.8 %, *n* = 106); and their average age was 22.5 years. Table 2 presents a result comparison between the different estimation methods. The first panel in Table 2 displays the total counts for the Best-Worst Scaling of our sample of 190 respondents; the second panel contains individual-level scores. For each panel, the first two columns indicate the number of times the specific specialty was selected as the most/least important for all of the respondents. Next to the columns, the BW scores are calculated by the best score minus the worst score for the specialty. General surgery (which scored 416) and internal medicine (which scored 320) have been identified as the most popular medical specialties among our sample; paediatrics, as a PCP-relevant specialty, has also been deemed as a preferred choice; while both psychiatry and geriatric medicine were the least preferred specialties according to the results.

**Table 2:** Results comparison between the different estimation methods

Factors	Total counts				Individual proportion					Conditional logit	Shares of preferences	K-means clustering	
	Most attractive	Least attractive	BW scores	Standardised BW	Most attractive	Least attractive	BW scores	Standardised BW	SD			Cluster 1	Cluster 2
Internal medicine	364	44	320	0.337	1.916	0.232	1.684	0.337	0.397	0.955	0.182	2.083	1.159
General surgery	481	65	416	0.438	2.532	0.342	2.190	0.438	0.469	1.209	0.235	1.398	3.232
Ob & Gyn	277	153	124	0.131	1.458	0.805	0.653	0.131	0.516	0.396	0.104	1.472	−0.427
Paediatrics	251	173	78	0.082	1.321	0.911	0.411	0.082	0.527	0.303	0.095	1.907	−1.561
Ophthalmology	174	113	61	0.064	0.916	0.595	0.321	0.064	0.354	0.246	0.090	0.194	0.488
Otolaryngology	112	208	−96	−0.101	0.590	1.095	−0.505	−0.101	0.423	−0.219	0.056	−0.898	0.012
Oncology	151	197	−46	−0.048	0.795	1.037	−0.242	−0.048	0.422	−0.079	0.065	−1.352	1.220
Geriatric medicine	21	266	−245	−0.258	0.111	1.400	−1.290	−0.258	0.315	−0.644	0.037	−0.963	−1.720
Dermatology	70	313	−243	−0.256	0.368	1.647	−1.279	−0.256	0.421	−0.624	0.038	−1.611	−0.841
Psychiatry	53	403	−350	−0.368	0.279	2.121	−1.842	−0.368	0.420	−0.922	0.028	−2.250	−1.305
Rehabilitation	136	155	−19	−0.020	0.716	0.816	−0.100	−0.020	0.399	*0.000*	0.070	0.019	−0.256
sum	-	-	-	-	-	-	-	-	-	-	1.000	-	-

Note: the number in italic style is the baseline level for conditional logit

**Fig. 1:**
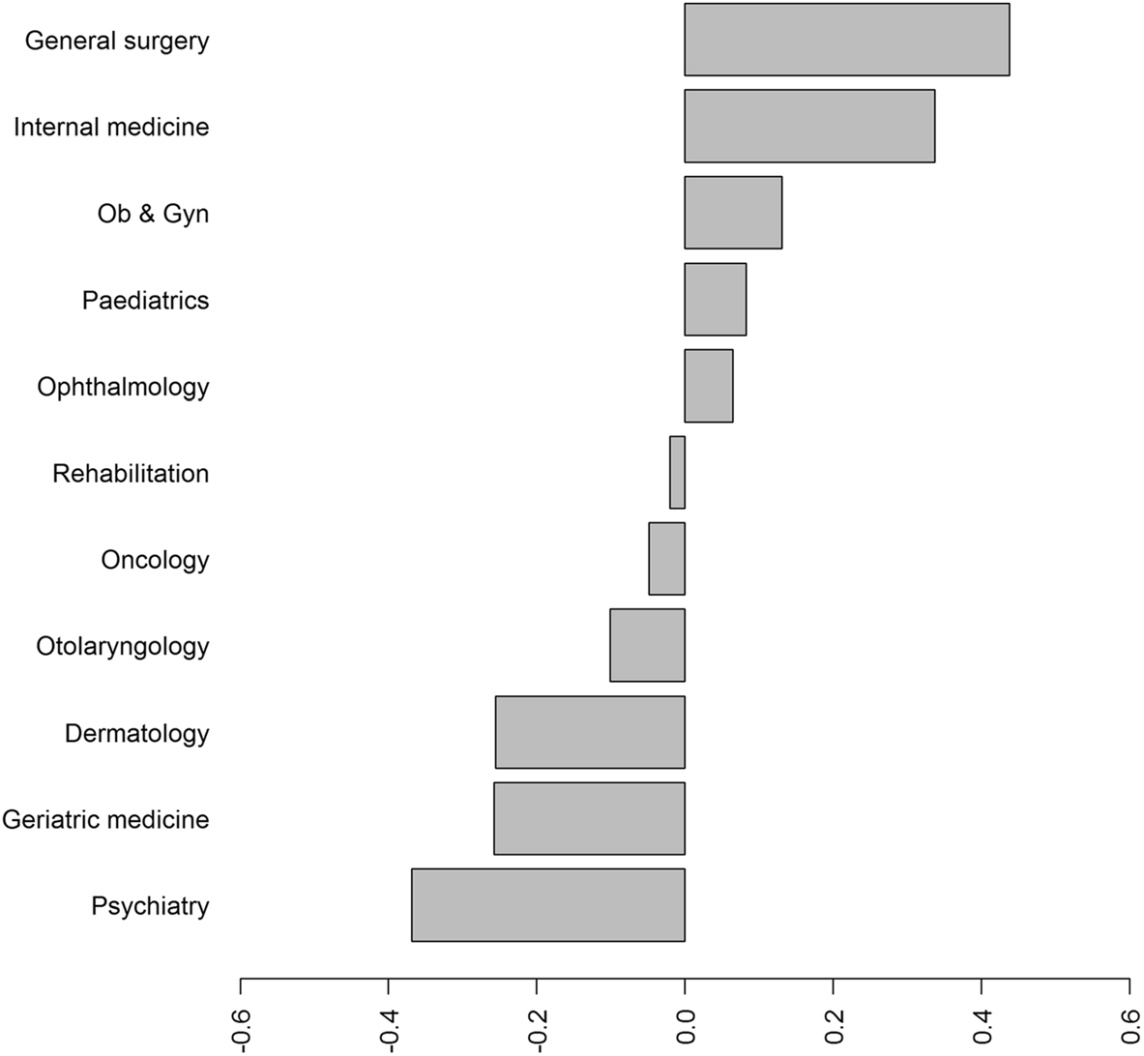
Standard BW scores for the specialty on registration for organ donation

In order to compare the relative importance of all of the medical specialties listed, we ordered the mean of the standardised BW scores and plotted them in Fig. 1. The figure shows consistent results as in Table 2, in which the most important item in the respondents’ decision is still general surgery; however, the respondents put almost the same weight of preference on internal medicine. In total, six specialties – (rehabilitation, oncology, otolaryngology, dermatology, geriatric medicine and psychiatry) were not among the preferred specialties.

**Fig. 2:**
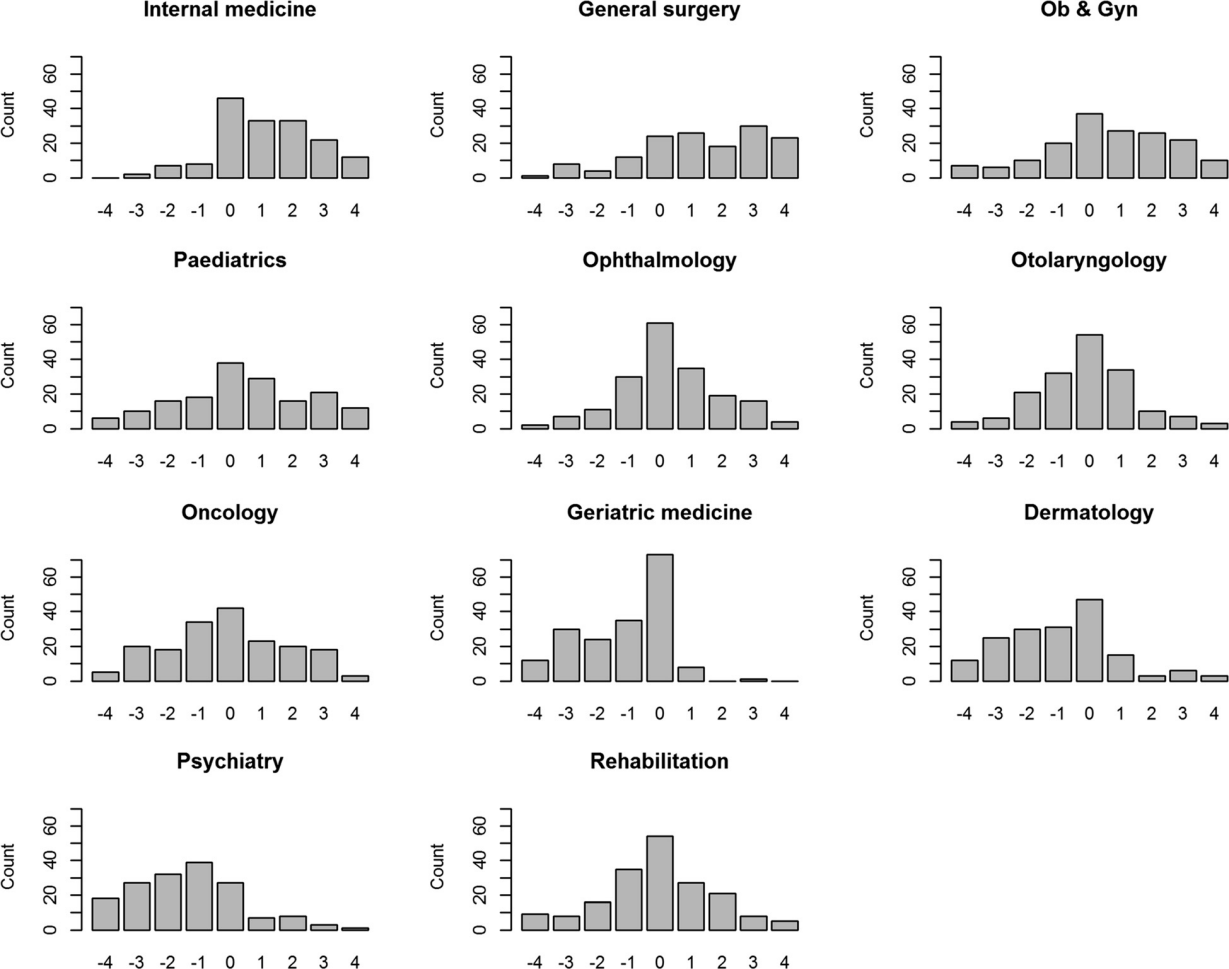
Distributions of BW scores by specialty

Figure 2 illustrates the distribution of full BW scores by their specialty based on individual-level BW scores to explore the heterogeneity of preference across this sample. To plot this figure by each specialty, we first transformed the BW scores into an integer ranging from −4 to 4, then we calculated the number of respondents for each integer. Figure 2 demonstrates that some distributions, such as internal medicine and geriatric medicine, reflected the violation of normality assumption, thus implying the existence of different types of specialty preferences for the respondents.

**Fig. 3:**
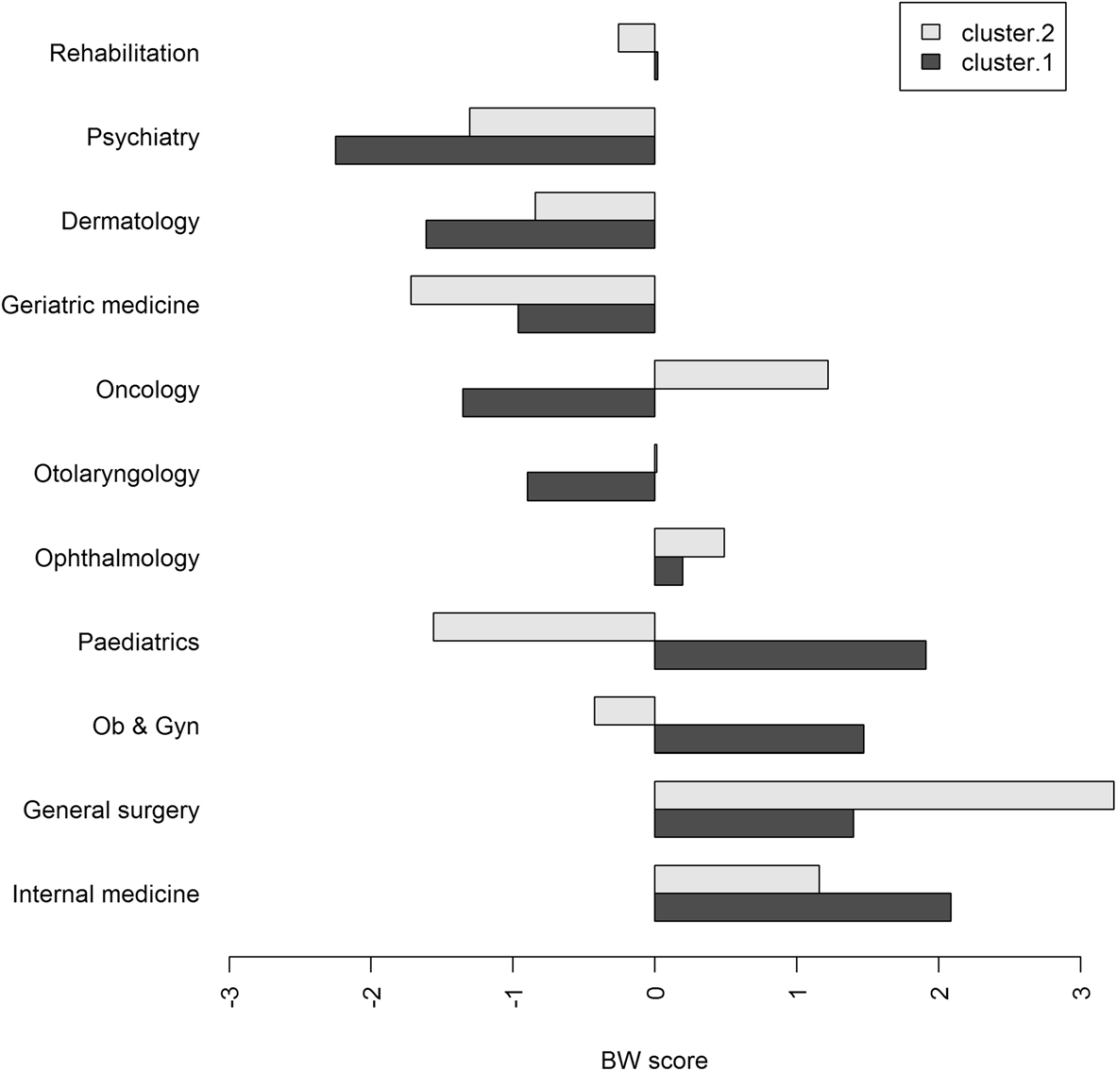
Mean BW scores per specialty in two clusters

The last two columns in Table 2 present the results of the K-means clustering. This sample is classified into two groups with different medical specialty selection preferences. According to the estimates from the K-means clustering, we further draw the mean BW scores by specialty into two clusters in Fig. 3. We observed relatively large differences in the BW scores between the two groups with respect to paediatrics, oncology and obstetrics/gynaecology.

Finally, Table 2 reports the result comparison between the different estimation methods. This table includes standardised BW scores based on a counting approach in the first results column. The coefficients of the conditional logit model are fundamentally consistent with the results of the first panel – the counting statistics. General surgery and internal medicine are confirmed as the most popular specialties for graduating medical students selecting their future career. The calculated share of preferences based on conditional logit estimates is also consistent with those results from the counting method.

## Discussion

In this study, we use a case 1 BWS experiment to study the preference for medical specialty selection among a sample of graduating Chinese medical students. To our knowledge, there are no previous papers utilising a case 1 BWS method to identify medical students’ specialty choices, especially in China’s unique context. However, previous work has also described how important graduating medical students’ perceptions and attitudes towards their specialty choice can be [22–24]. Our study assigned a preference priority to eleven medical specialties deemed most-cited alternatives when graduating medical students select their future career.

However, China has a special background in education for health professionals that needs some clarification before the results are interpreted further. Like many other countries, the majority of students in high schools are admitted into medical colleges or universities through a nationwide college entrance examination. The difference in China is that medical students are often assigned based on their exam scores rather than their expressed first choice career preference. In addition, the medical training or residency programmes after graduating from medical school are an extremely important part of becoming a “real” doctor in many developed countries. This postgraduate medical training is a well-established system integrating hospitals, medical schools, and relevant medical associations. Nonetheless, China has introduced a similar pilot training model after 2003. The training programme spread throughout the whole nation after 2010. Finally yet importantly, general practice or family medicine, as the foundation of PCP, has developed as an academic discipline in universities since 2011 [18]. Therefore, we have to follow the previous classification and use internal medicine, paediatrics and geriatric medicine as the PCP-relevant specialties [6, 19].

In the context of this background, we found that general surgery, a non-PCPS specialty, was identified as the most preferred specialty among the overall sample. Internal medicine was of almost the same importance as surgery. Obstetrics/gynaecology and paediatrics were also among the most preferred specialties.

As the results show, two out of three PCP-relevant specialties have been given the most weight by graduating medical students. However, both geriatric and psychiatric medicine were found to be the least selected, showing that geriatric medicine, as one of PCP-relevant specialties, is not viewed as a popular specialty.

In comparison with previous case studies in other countries, this study is in line with a study in Jordan in which a survey showed that surgery, internal medicine, paediatrics, and obstetrics/gynaecology were the most preferred specialties among medical students. Another study in Canada found medical students’ most popular choices were internal medicine and associated subspecialties, surgery and associated subspecialties, paediatrics and family medicine. Moreover, students at a medical school in Japan were most interested in internal medicine, general surgery, paediatrics, and emergency medicine [17, 25, 26].

Furthermore, the strengths of this study have been reinforced by its novel methodology. The superiority of BWS over typical preference measurement instruments is clear. 5-point or 7-point Likert rating scales have been observed to be vulnerable to some critical challenges. For example, the respondents tend not to discriminate, or make trade-offs in economics, between items when they are asked to rate the importance of each item (specialty). In a BWS experiment, the observed choice frequencies ensure that the derived numbers are on a known frequency or the probability scale, avoiding inferring preference strength by asking respondents to use a scale number. Thus, BWS can be used for comparison across different countries, rather than being vulnerable to different scale issues when various groups of people rate the same items. The BWS approach also avoids the issue of a large number of choice questions, such as paired comparisons, because BWS applies a statistical design to construct the choice sets.

The other advantage of the present study is that it has examined the impact of preference heterogeneity on the estimates through a counting approach, a conditional logit model, and K-means clustering. The K-means clustering approach has revealed substantial preference heterogeneity across the respondents, which suggests that the policy makers need to pay attention to different groups of medical students.

We have to acknowledge that a couple of factors limit this study. First, we restricted our sample in the survey to include only medical students in three colleges, which may threaten the external generalisation of our results. However, it is reasonable to argue that we surveyed graduating medical students, who are conceived to be more likely to make the final decision on specialty selection than medical students in lower grades. Second, the study may suffer from several caveats to which any BWS study would be vulnerable. For instance, the varying degrees of similarity between the items are hard to examine; more specifically, the respondents cannot report “none of the specialties are good/bad.” In addition, the BWS experiment was designed to examine the students’ preferences and perceptions instead of their real decisions.

## Conclusion

In conclusion, this study is the first to apply a Best-Worst Scaling method to elicit graduating medical students’ preferences for their future career specialty. We found that despite the fact that a non-PCP-based specialty was the most preferred selection among sampled students, a PCP-based specialty, such as internal medicine, was still a popular alternative among our sample in China. Given the evidence of a strong relationship between the students’ preferences and future choices for a medical specialty, this study contributes to a better understanding of the career desires of medical students, and thus the future structure of the health care workforce. We cannot draw a conclusion that medical students in China prefer PCP-relevant specialties; especially general practice, as China has launched a number of new policies aimed at cultivating general practitioners with a target number of 300,000 by 2020. However, the results from our study can be used as a baseline measurement to assess the impact of health care and medical education reforms in primary care. Finally, this study can provide an insight into the provision of human resources for health in a hospital-dominated health care system like China.

## Appendix A

**Table:** ᅟ

Choice set	alt 1	alt 2	alt 3	alt 4	alt 5
No 1	3	4	6	9	10
No 2	2	5	6	8	9
No 3	1	4	8	9	11
No 4	1	3	5	8	10
No 5	5	7	9	10	11
No 6	1	2	3	7	9
No 7	2	4	7	8	10
No 8	3	6	7	8	11
No 9	1	2	6	10	11
No 10	1	4	5	6	7
No 11	2	3	4	5	11

Note: This is the Balanced Incomplete Block Design (BIBD) matrix for the survey questionnaire. The first column indicates the numerical order of the choice set; each choice set includes five alternatives; the simple number values respectively correspond to the 11 specialties for medical students’ choice

## Abbreviations

BIBD: balanced incomplete blocked design
BWS: best-worst scaling
CL: conditional logit
PCP: primary care providers
